# Characterization of the Gastric Antrum Microbiome in *Helicobacter pylori*-Negative Individuals: Insights from a Greek Population Using 16S rRNA Next-Generation Sequencing

**DOI:** 10.3390/pathogens15030290

**Published:** 2026-03-06

**Authors:** Asimoula Kavvada, Georgia Gioula, Andreas Protopapas, Adonis A. Protopapas, Maria Christoforidi, Fani Minti, Christos Savopoulos, Maria Chatzidimitriou

**Affiliations:** 1 Department of Biomedical Sciences, International Hellenic University, 57400 Thessaloniki, Greece; 2 Department of Microbiology, Medical School, Aristotle University of Thessaloniki, 54124 Thessaloniki, Greece; 3 First Propaedeutic Department of Internal Medicine, AHEPA University Hospital, Aristotle University of Thessaloniki, 54636 Thessaloniki, Greece

**Keywords:** gastric microbiome, *Helicobacter pylori*, 16S rRNA, next-generation sequencing, antrum

## Abstract

Background: Once considered a sterile organ, the human stomach is now known to harbor a diverse microbial community that may influence both gastric homeostasis and disease. While extensive research has been conducted worldwide, regional variation in the gastric microbiome remains insufficiently characterized. This study aimed to describe the gastric antrum microbiome of *Helicobacter pylori*-negative Greek adults using 16S rRNA next-generation sequencing (NGS). Methods: Samples of gastric biopsies were obtained from patients undergoing gastroscopy at a tertiary hospital in Greece. *H. pylori* infection was excluded through a combination of bacterial culture and patient medical history. The final study group consisted of 9 subjects. Following DNA extraction, the 16S rRNA gene was sequenced on the Ion Torrent™ platform. Bioinformatic processing and statistical analyses were performed using the phyloseq, vegan, and ggplot2 R packages. Microbial composition, relative abundance, and alpha diversity (Shannon and Inverse Simpson indices) were evaluated at the genus level. Results: The gastric microbiome comprised 19 phyla, 150 families, 213 genera, and 391 species. The predominant phyla were Proteobacteria (36.92%), Firmicutes (34.21%), and Bacteroidetes (12.97%). The most prevalent families were *Streptococcaceae*, *Helicobacteraceae*, *Prevotellaceae*, and *Pasteurellaceae*. At the genus level, *Streptococcus* (21.71%), *Helicobacter* (18.39%), and *Prevotella* (9.99%) accounted for nearly half of the total relative abundance. Alpha diversity indices indicated moderate richness and evenness across samples. Conclusions: The gastric antrum microbiome of *H. pylori*-negative Greek individuals exhibits substantial taxonomic diversity dominated by Proteobacteria and Firmicutes. The microbial community structure aligns closely with profiles reported in other global populations. These findings provide a reference baseline for future comparative analyses involving *H. pylori*-positive individuals to better understand microbiome shifts associated with colonization and gastric disease.

## 1. Introduction

The human microbiome consists of an enormous community of approximately 10 to 100 trillion symbiotic microbial cells, each carrying its own set of genes [1,2]. The development of non-culture-based molecular methods has revolutionized the study of these microbial ecosystems. Among these approaches, next-generation sequencing (NGS) has become a defining tool, with 16S rRNA gene sequencing serving as a cornerstone for decoding the complexity and dynamics of the human microbiome [3,4,5,6].

The human microbiome is distinguished by two fundamental features. First, it displays striking diversity, both between individuals and across distinct anatomical niches within the same host. Second, it shows considerable plasticity, with microbial composition capable of shifting in response to internal and external influences such as diet, medications, environment, and disease state, although the degree of this plasticity varies by body site [7]. The composition and functionality of the microbiome play a critical role in human health, influencing processes that range from metabolism and immune regulation to neurological function. Detailed characterization of the microbiome and its functional variants has the potential to significantly advance diagnostic precision, therapeutic development, and preventive medicine. In recent years, international projects have been developed to investigate both the diversity and the functional potential of the microbiome. Representative examples are the Human Microbiome Project (HMP) and the MetaHIT (Metagenomics of the Human Intestinal Tract) projects, collaborative initiatives aimed at characterizing human gut microbial genes and associated with human homeostasis and diseases [8,9].

The gastrointestinal tract represents one of the most densely colonized microbial habitats, where microbial communities perform a broad range of essential physiological functions, including providing a natural barrier against pathogenic colonization, contributing to nutrient digestion and drug metabolism, modulating host immune responses, and maintaining systemic metabolic and immunological homeostasis [10,11,12,13].

Historically, the stomach was considered a sterile environment due to its acidic conditions, until 1982, when Marshall and Warren overturned this paradigm with the discovery of *Helicobacter pylori* [14]. Since then, the stomach has been recognized as hosting a distinct microbial ecosystem [15,16].

Bik and colleagues were the first to apply non-culture-based sequencing to gastric mucosal samples [15]. Subsequent 16S rRNA-based studies have confirmed that Firmicutes, Bacteroidetes, Proteobacteria, and Actinobacteria represent the principal phyla in the gastric microbiome, with *Streptococcus* typically emerging as the most dominant genus, followed by *Prevotella* and *Veillonella* [16,17,18,19,20,21,22,23,24,25,26,27,28,29].

Although significant progress has been made in characterizing the human gastric microbiome through NGS approaches, many aspects remain insufficiently understood. Published findings regarding the distribution, relative abundance, and functional role of microbial communities across gastric regions remain inconsistent, with some studies reporting significant differences between the antrum and corpus and others finding little to no variation [16,20,21].

In this context, the present study aimed to characterize the antrum microbiome of *Helicobacter pylori*-negative Greek adult patients undergoing gastroscopy at a tertiary hospital in Greece. By analyzing both the taxonomic composition and relative abundance of bacterial communities, we sought to:

- Evaluate the microbial diversity of the gastric antrum.
- Compare our findings with previously published international data, thereby providing new reference evidence from a Greek patient cohort.

It is worth noting that the present study represents the first experimental investigation of the gastric microbiome in Greece, employing next-generation sequencing techniques. An additional innovation lies in the exclusive focus on the antrum region of the stomach.

## 2. Materials and Methods

Study Population. The study population consisted of 9 adult participants (>17 years) of both sexes and of Greek nationality. No upper age limit was applied. The following exclusion criteria were implemented:

1. Recent use of proton pump inhibitors (PPIs);
2. Current or recent use of H2 receptor antagonists or other antacid medications;
3. Use of probiotic supplements;
4. Use of gastroprotective agents;
5. Administration of antibiotics within one month prior to endoscopy;
6. History of total or partial gastrectomy;
7. Current *H. pylori* infection;
8. Detection of *H. pylori* based on conventional methods (gastric biopsy culture);
9. Eradication therapy for *H. pylori* infection in the past.

These criteria were selected because each of the listed factors is known to significantly influence or disrupt the native composition of the gastric microbiome [30]. The identification of *H. pylori* was performed through the culture of biopsy samples obtained from the gastric body and antrum. A brief medical history was collected from each patient to determine exclusion criteria (Appendix S1—Supplementary Materials).

Females accounted for 66.6% of the study population, while males represented 33.3% (six females and three males, respectively). The average age of the participants was 58.8 years old, while 8 to 9 individuals were over 50 years old. None of the participants reported excessive consumption of alcohol, and only one participant was indicated as a smoker. Clinically, none of the participants exhibited signs or symptoms of acute gastritis, nor did they report a history of *H. pylori* infection or gastritis. However, endoscopic evaluation revealed mild antral erythema in several cases.

Data Collection and Patient History. A brief medical history was obtained from each participant, including demographic information (sex, place of origin, age), lifestyle factors (smoking status and alcohol consumption), current symptoms and clinical presentation, and detailed medication use, with particular attention to drugs relevant to the exclusion criteria. Information was also obtained regarding the known history of *H. pylori* infection and treatment—both personal and within the family environment—as well as potential complications related to infection, including peptic ulcer disease, gastric cancer, or other gastrointestinal disorders.

Ethical Approval and Consent. All participants received written information regarding the study (Appendix S2—Supplementary Materials) and signed informed consent forms (Appendix S3—Supplementary Materials), in accordance with the requirements of the Institutional Bioethics Committee.

Sample Collection. Gastric biopsy specimens were obtained during scheduled gastroscopies performed at the tertiary hospital. Four biopsies were collected from each participant: three for *H. pylori* detection and one for microbiome analysis via NGS. For *H. pylori* identification, biopsies were obtained following the Sydney protocol. For microbiome sequencing, a single biopsy specimen was collected from the gastric antrum.

Sample Handling and Storage. Biopsy specimens intended for *H. pylori* culture were placed in specialized anaerobic transport medium and delivered to the microbiology laboratory within four hours of collection. Biopsies designated for sequencing were preserved in sterile, pyrogen-free, DNase- and RNase-free cryotubes made of polypropylene, resistant to temperatures down to −196 °C. These samples were immediately stored at −70 °C until further processing.

*H. pylori* identification. Bacterial cultures were incubated under microaerophilic conditions (5% O_2_, 10% CO_2_, and 10% H_2_), established using the GasPak™ EZ pouch system (BD, Canada), and maintained at 37 °C for 76 h in a dedicated incubation chamber. Final identification of *H. pylori* colonies was based on colony morphology, microscopic examination of Gram-stained smears, and biochemical testing for catalase and oxidase enzymatic activity, both characteristic of *H. pylori*.

Histological examination of gastric biopsy specimens was performed for all participants, in accordance with the clinical indications of the attending gastroenterologists. The results were disclosed to the investigators following the participants’ informed consent.

DNA Extraction. Genomic DNA was extracted from gastric biopsy samples using the DNeasy PowerSoil Pro Kit (QIAGEN, Hilden, Germany), following the manufacturer’s protocol [31]. DNA purity and concentration were assessed spectrophotometrically using a NanoDrop™ One (Thermo Fisher Scientific, Carlsbad, CA, USA), and integrity was confirmed by agarose gel electrophoresis [32].

Library Preparation and NGS. Library preparation was performed with the Ion Plus Fragment Library Kit (Thermo Fisher Scientific Inc., Waltham, MA, USA). Sequencing was carried out on the Ion Torrent™ Sequencing System using the Ion 510™, Ion 520™, and Ion 530™ Kit—Chef (Thermo Fisher Scientific Inc., Life Technologies Corporation, Carlsbad, CA, USA), according to the manufacturer’s instructions. The sequencing workflow targeted the V3–V4 hypervariable regions of the 16S rRNA gene. Raw sequence data were subjected to quality control filtering, trimming, and chimera removal prior to downstream analyses [32,33].

Statistical and Diversity Analysis. All bioinformatic and statistical analyses were performed in R (version 3.5.2; R Core Team, New Zealand, Australia). The R packages vegan (v2.5.3), phyloseq (v1.24.2), and ggplot2 (v3.1.0) were used for data processing and visualization. For Relative Abundance Analysis, statistical significance was set at *p* < 0.05. Visualizations included bar plots and heatmaps. Within-sample (alpha) diversity was evaluated at the genus level using the Shannon and Inverse Simpson indices [34,35,36,37,38,39,40,41].

## 3. Results

Regarding demographic and clinical characteristics, nine individuals participated in the study, including six females and three males. The majority of participants (8/9) were over 50 years of age, with a mean age of 58.8 years. None reported excessive alcohol consumption, only one participant was identified as a current smoker, and one participant reported adherence to a vegetarian diet. Clinically, none of the participants exhibited signs or symptoms of acute gastritis, nor did they report a prior history of *H. pylori* infection or gastritis. However, endoscopic evaluation revealed mild antral erythema in six of the nine participants. None of the participants reported the use of antibiotics, proton pump inhibitors (PPIs), H2 receptor antagonists, other antacid medications, probiotic supplements, or gastroprotective agents within at least two months prior to sampling. Four out of nine participants were receiving chronic medication for thyroid disorders, diabetes mellitus, or cardiovascular conditions. Only one participant reported a history of surgically treated gastric gastrointestinal stromal tumor (GIST), while the remaining participants had no significant medical history.

In total, 3,098,734 reads were recorded across all taxonomic levels and samples. A total of 924,891 reads were detected at the phylum level, 924,891 at the family level, 785,938 at the genus level, and 463,014 at the species level. The total number of taxa identified across all taxonomic levels amounted to 773. Specifically, 19 phyla, 150 families, 213 genera, and 391 species were reported. Table 1 lists the ten most prevalent phyla, families, genera, and species based on relative abundance.

Regarding relative abundance, the three predominant phyla were Proteobacteria, Firmicutes, and Bacteroidetes, collectively representing over 80% of the total microbial composition. Particularly, Proteobacteria accounted for 36.92%, Firmicutes for 34.21%, and Bacteroidetes for 12.97%. These are followed by the phyla Actinobacteria (9.52%), Fusobacteria (4.07%), and Cyanobacteria (2.19%), with the combined abundance of these six phyla accounting for 99.88% of the total.

**Figure 1:**
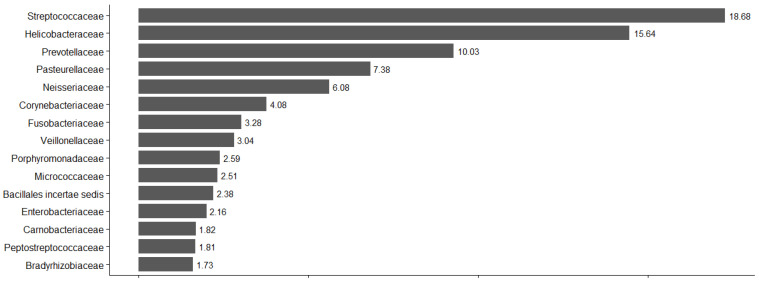
Abundance of the top 10 families of the total study population.

Similarly, the four most abundant families represented approximately 50% of the total microbial abundance. The *Streptococcaceae* family exhibited the highest relative abundance (18.68%), followed by *Helicobacteraceae* (15.64%), *Prevotellaceae* (10.03%), and *Pasteurellaceae* (7.38%). The relative abundance of the 15 prevalent families represented 89.82% of the total relative abundance and is demonstrated in Figure 1.

**Figure 2:**
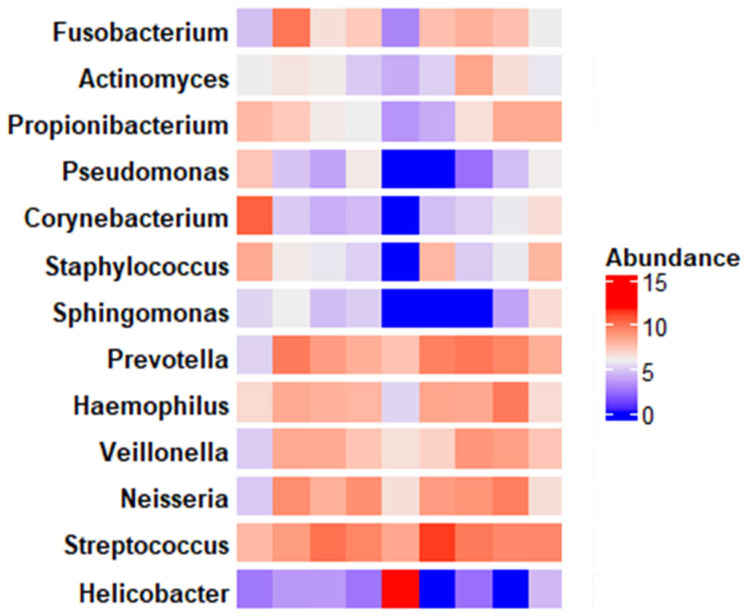
Abundance of the genera belonging to the predominant families across all specimens.

At the genus level, we identified *Streptococcus* (21.71%), *Helicobacter* (18.39%), and *Prevotella* (9.99%). The relative abundance of the 15 prevalent genera accounted for roughly 50% of the total abundance. Figure 2 illustrates the abundance of the genera belonging to the predominant families across all specimens.

**Figure 3:**
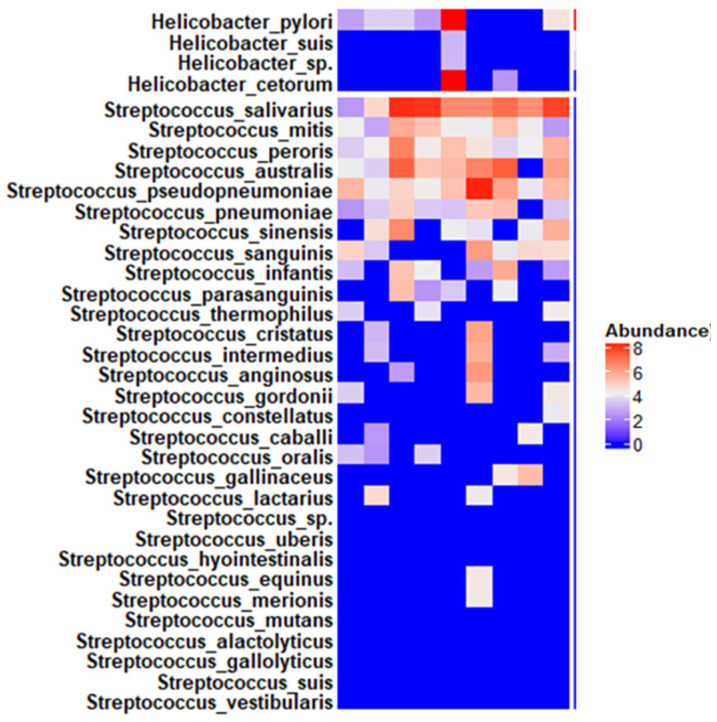
Abundance of the species belonging to the two predominant genera (*Streptococcus* and *Helicobacter*) across all specimens.

Finally, *Helicobacter pylori* (23.41%), *Prevotella melaninogenica* (7.54%), and *Fusobacterium periodonticum* (4.43) were the three predominant species. The relative abundance of the 20 prevalent species represented 73.1% in total. Figure 3 demonstrates the abundance of the species belonging to the two predominant genera (*Streptococcus* and *Helicobacter*) across all specimens. Additional raw data on taxonomic abundance at all levels and species-level abundance per sample are available in the Supplementary Materials Section (Supplementary Files S2 and S3).

**Figure 4:**
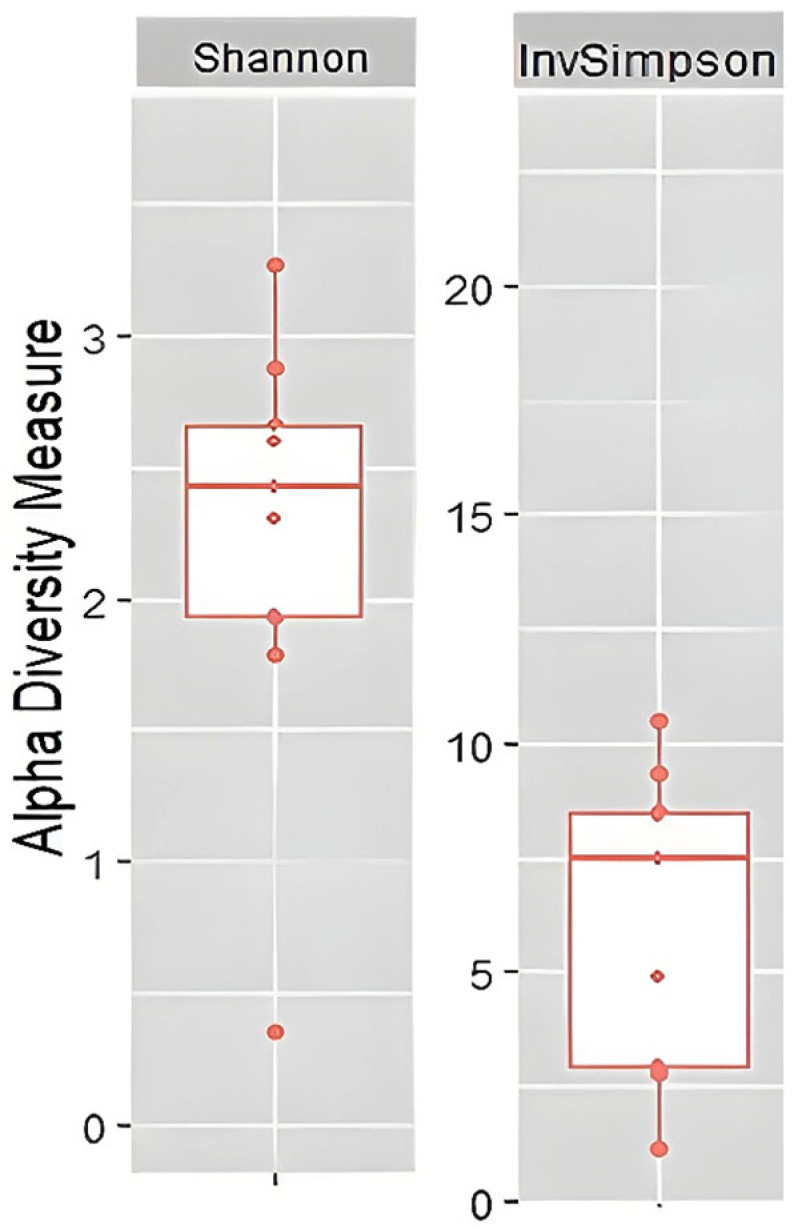
Inverse Simpson and Shannon indices, calculated at the genus level. The red line within each box indicates the median value and represents the central tendency of the distribution. Median Shannon index (**left**) = 2.4. Median Inverse Simpson index (**right**) = 7.4.

To assess alpha diversity, the Inverse Simpson and Shannon indices were calculated at the genus level [42,43,44,45,46]. The median Inverse Simpson index was 7.4, while the median Shannon index was 2.4 (Figure 4). Both indices indicate moderate to high richness and an evenness of genera within our study group. Samples S14 and S24 exhibit the highest microbial diversity, with the Shannon index recorded to 3.26 and 2.88, and the Inverse Simpson index amounted to 10.51 and 9.34, respectively. Sample S10 stands out markedly from the others, as its considerably lower index values (Shannon 0.36 and Inverse Simpson 1.14) indicate a low richness of microbiota. Additional data on α-diversity indices are available in the Supplementary Materials Section (Supplementary File S4).

## 4. Discussion

Our study represents the first investigation in Greece to characterize the gastric microbiome of the antrum using next-generation sequencing techniques. According to our results, the most abundant phyla were Proteobacteria and Firmicutes, followed by Bacteroidetes, Actinobacteria, Fusobacteria and Cyanobacteria. At the genus level, *Streptococcus*, *Helicobacter*, and *Prevotella* together accounted for roughly 50% of total abundance, followed by *Neisseria*, *Corynebacterium*, and *Haemophilus*. The three predominant species were *Helicobacter pylori*, *Prevotella melaninogenica* and *Fusobacterium periodonticum*. Despite *H. pylori* being the most abundant species, 108,233 of the 108,411 reads were detected in a single sample, belonging to a 70-year-old female participant, a non-smoker, with a recent history of esophagitis. Similarly, *Helicobacter cetorum*, although ranked 8th in relative abundance, was exclusively found in the same participant. No other species belonging to the *Helicobacter* family was detected in the study sample. Moreover, the relatively high alpha diversity indicates a robust microbial community, with relatively high evenness and richness within our study group.

To date, culture-independent methodologies have gradually replaced conventional methods, giving a better insight into the gastric bacterial community profile. Available evidence suggests that, under physiological conditions, gastric microbiota is primarily composed of members of the phyla Actinobacteria, Bacteroidetes, Firmicutes, and Proteobacteria. At the genus level, *Streptococcus* is consistently reported as one of the most prevalent taxa [47,48].

As was referred to above, the most abundant bacterial phyla that dominate the gastric antrum in our study group were Proteobacteria, Firmicutes, Bacteroidetes and Actinobacteria. These results are consistent with previous studies. For example, Bik et al. (2006) analyzed 23 healthy Caucasian individuals and identified Firmicutes, Proteobacteria, and Actinobacteria as the dominant phyla. Notably, the researchers also detected members of the phylum Deinococcota, previously known to inhabit extreme environments such as radioactive waste sites, but never before reported in humans. This phylum was also identified at low abundance in our study group [15]. Similarly, Anderson et al. (2008) conducted a comparative analysis of the microbiota of throat, stomach and fecal samples by Barcoded Pyrosequencing. Gastric samples were gathered from 6 subjects—3 negative and 3 positive samples for *H. pylori* according to culturing. The most abundant phylum in *H. pylori* negative samples was Actinobacteria, followed by Firmicutes, Bacteroidetes and Proteobacteria [49]. Delgado et al. (2013) reported Firmicutes and Proteobacteria as the predominant phyla in 12 Spanish participants, while in a cohort of 84 *H. pylori*-negative Malaysian residents, Khosravi et al. (2014) identified three predominant phyla: Proteobacteria, followed by Firmicutes and, to a lesser extent, Actinobacteria [17,50]. Ndegwa et al. (2020) detected Firmicutes, Bacteroidetes, Proteobacteria, Actinobacteria, and Fusobacteria in 171 Swedish participants negative for *H. pylori* [19]. In addition, Lopetuso et al. (2014)—in 4 *H. pylori*-negative Ameridians—and Huang et al. (2024)—in a cohort of 14 Chinese participants—found Firmicutes, Bacteroidetes, Actinobacteria, and Proteobacteria to be the most prevalent phyla [20,51]. Overall, phylum-level microbial patterns appear largely conserved across different populations. However, in our study, the relatively high predominance of Proteobacteria was likely influenced by the exceptionally high abundance of *H. pylori* observed in a single participant.

At the family level, the elevated presence of Proteobacteria in our cohort, was mainly attributable to *Helicobacteraceae*, while families within Firmicutes and Bacteroidetes also contributed substantially to microbial composition. Comparable patterns were observed in other studies: Bik et al. (2006) reported low-abundance families, which were similarly detected in our samples [15]. Delgado et al. (2013) noted a mixture of dominant and less abundant families, reflecting significant inter-individual variability [17]. Huang et al. (2024) also identified both dominant and low-abundance families in the 14 Chinese participants, illustrating geographic and ethnic differences at this taxonomic level [20].

At the genus level, *Streptococcus*, *Helicobacter*, and *Prevotella* were recorded as the predominant genera, followed by *Neisseria*, *Corynebacterium*, and *Haemophilus*. Bik et al. (2006) reported *Streptococcus* as the predominant genus, followed by *Prevotella*, *Rothia*, *Fusobacterium*, and *Veillonella* species [15]. Zilberstein et al. (2007) conducted a survey based on cultural methods and taxonomically identified the genera *Clostridium*, *Lactobacillus*, and *Veillonella* as the most abundant of the healthy human stomach [52]. According to Anderson et al. (2008), *Streptococcus*, *Actinomyces*, *Prevotella* and *Gemella* were the most abundant genera in the three *H. pylori*-negative cases. These genera were also abundant in the throat samples, suggesting a possible translocation of bacteria originating from the oral cavity [49]. Dicksved et al. (2009) observed *Streptococcus*, *Lactobacillus*, *Veillonella*, and *Prevotella* as dominant genera in five Swedish participants, with Helicobacter present at low abundance [18]. Delgado et al. (2013) identified *Lactobacillus*, *Streptococcus*, and *Propionibacterium* as prevalent genera in 12 Spanish participants; however, in our cohort these genera ranked lower in relative abundance (*Lactobacillus*, 18th; *Propionibacterium*, 14th) [17]. Khosravi et al. (2014) identified in 84 *H. pylori*-negative Malaysian participants Streptococci, Neisseria, Klebsiella, and Lactobacilli as the predominant genera [50]. In a larger cohort of 171 Swedish participants, Ndegwa et al. (2020) reported dominant genera including *Streptococcus*, *Prevotella*, *Veillonella*, *Fusobacterium*, *Haemophilus*, *Neisseria*, and *Gemella*, findings that are largely consistent with our results [19]. In contrast, Huang et al. (2024) identified *Brevundimonas*, *Helicobacter*, *Vibrio*, and *Pseudoalteromonas* as the dominant genera in 14 Chinese participants, highlighting geographic variability in gastric microbial composition [20]. Across studies, alpha diversity at the genus level was moderate to high: Ndegwa et al. (2020) reported a Shannon diversity index of 3.16, while Huang et al. (2024) reported a Chao1 index of approximately 120 and a Shannon index of approximately 3.8 [19,20].

Regarding regional differences in microbial composition or abundance within the stomach, findings across the studies have been inconsistent. For instance, Sohn et al. (2017) reported comparable bacterial loads between the gastric corpus and antrum. In two *H. pylori*—negative individuals, Proteobacteria dominated both regions, while Actinobacteria were present at lower relative abundancies. *Firmicutes* and *Streptophyta* were detected exclusively in corpus samples. In contrast, in two *H. pylori*-positive individuals, the pathogen appeared to outcompete and suppress the commensal microbiota across both gastric regions [21]. More recently, Huang et al. (2024) analyzed microbiota from antral biopsies only and reported that, in healthy individuals, the most abundant phyla (Firmicutes, Bacteroidetes, Actinobacteria, Proteobacteria) were consistent with previous reports. However, differences emerged at the genus level, with *Brevundimonas*, *Vibrio*, *Pseudoalteromonas*, *Aeromonas*, and *Prevotella* predominating [20]. Our findings are partially consistent with both studies, as Proteobacteria and Firmicutes emerged as the predominant phyla in the antral niche, while *Streptococcus*, *Helicobacter*, and *Prevotella* were identified as the most abundant genera.

Although the composition of our study cohort did not allow for the investigation of secondary variables that may influence the gastric microbiome, the international literature provides evidence regarding the impact of multiple hosts and environmental and clinical factors on gastric microbial composition.

Differences in the gastric microbiome between individuals of different ethnic backgrounds or regions may partly relate to dietary habits [10]. For example, a diet high in salt primarily alters the composition of the gastric microbiota by reducing the relative abundance of Bacteroidetes and Proteobacteria at the phylum level and lowering the abundance of Unclassified_S24-7 and Lactobacillus at the genus level [53].

Age and sex are controversial factors influencing the gastric microbiome. A long-term follow-up study of individuals not colonized by *H*. *pylori* and without reported atrophic gastritis or intestinal metaplasia showed that microbial diversity, as well as the abundance of Firmicutes and Fusobacteria, decreased over time, while the abundance of Proteobacteria increased with age [27]. However, Li et al. (2017) reported that neither age nor sex significantly influenced the bacterial composition of the stomach [25].

To date, scientific data regarding the effects of conventional cigarette smoking on the gastric microbiome remain scarce. Nevertheless, a substantial body of evidence demonstrates that smoking adversely affects the composition and abundance of the gut microbiome. Toxic by-products of cigarette smoke constituents, as well as nicotine itself, can induce microbial dysbiosis, which has been associated with unfavorable clinical outcomes, including inflammatory bowel disease (IBD) and colorectal cancer [28].

Overall, the abundance of the gastric microbiome under physiological conditions appears to vary according to geographic latitude and ethnicity, whereas its taxonomic composition seems to remain relatively stable across populations. A notable finding in our study was the unexpectedly high abundance of *Helicobacteraceae*, *Helicobacter*, and *H*. *pylori*, which ranked as the second most abundant family, genus, and the dominant species, respectively. Importantly, however, all *Helicobacter* species were detected almost exclusively in a single sample. This apparent discrepancy likely reflects methodological limitations in detecting low-abundance taxa rather than true biological dominance. Supporting this interpretation, *Helicobacter* reads were overwhelmingly derived from a single participant. A similar phenomenon was reported by Bik et al. (2006), who suggested that the detection of *H. pylori* in apparently uninfected individuals may result from minor cross-contamination during DNA extraction or from the limited sensitivity of conventional diagnostic methods [15].

Limitations. Several limitations of the present study should be acknowledged. First, the relatively small sample size limited our ability to investigate the influence of secondary variables—such as age, sex, smoking status, dietary habits, medication use (including proton pump inhibitors and antibiotics), and comorbidities—on the gastric microbiome. Second, sampling was restricted to the antral region, precluding direct comparisons across distinct gastric niches, such as the corpus or fundus, which may harbor region-specific microbial communities. Finally, while next-generation sequencing enables sensitive detection of low-abundance taxa, it is also susceptible to technical biases, including low-biomass contamination and overrepresentation of taxa derived from individual samples, as observed for *Helicobacter* in this study. Moreover, taxonomic profiling alone does not provide insight into microbial function, which requires complementary metagenomic or transcriptomic approaches.

## 5. Conclusions

In conclusion, our findings reaffirm the fact that the gastric microbiome represents a diverse and dynamic microbial ecosystem. The application of NGS technology enables a more comprehensive and accurate characterization of this complex microbial community compared to conventional culture-based methods, which frequently underestimate bacterial diversity. The persistence of *Helicobacter* sequences in culture-negative samples underscores both the high sensitivity of molecular approaches and the possibility of low-level colonization or persistence of residual DNA. A deeper understanding of the taxonomic architecture and functional potential of the gastric microbiota, and of how these features are modulated by *H. pylori*, may yield novel insights into the mechanisms governing gastric homeostasis, disease pathogenesis, and host–microbe interplay within the upper gastrointestinal tract. Further studies integrating metagenomic, transcriptomic, and metabolomic approaches are warranted to clarify the biological significance of these microbial alterations and their implications for gastric physiology and pathology.

## Figures and Tables

**Table 1:** The predominant phyla, families, genera and species of the total study population.

Phylum	Reads	%	Family	Reads	%	Genus	Reads	%	Species	Reads	%
Proteobacteria	341,466	36.92	Streptococcaceae	172,789	18.68	Streptococcus	170,629	21.71	Helicobacter_pylori	108,411	23.41
Firmicutes	316,382	34.21	Helicobacteraceae	144,642	15.64	Helicobacter	144,498	18.39	Prevotella_melaninogenica	34,913	7.54
Bacteroidetes	119,989	12.97	Prevotellaceae	92,795	10.03	Prevotella	78,491	9.99	Fusobacterium_periodonticum	20,528	4.43
Actinobacteria	88,076	9.52	Pasteurellaceae	68,276	7.38	Neisseria	53,290	6.78	Haemophilus_parainfluenzae	20,343	4.39
Fusobacteria	37,685	4.07	Neisseriaceae	56,212	6.08	Corynebacterium	37,591	4.78	Rothia_mucilaginosa	17,979	3.88
Cyanobacteria	20,250	2.19	Corynebacteriaceae	37,714	4.08	Haemophilus	37,453	4.77	Neisseria_flavescens	17,071	3.69
Chloroflexi	245	0.03	Fusobacteriaceae	30,293	3.28	Fusobacterium	29,472	3.75	Propionibacterium_acnes	12,762	2.76
Tenericutes	226	0.02	Veillonellaceae	28,108	3.04	Veillonella	26,106	3.32	Helicobacter_cetorum	12,406	2.68
Planctomycetes	159	0.02	Porphyromonadaceae	23,973	2.59	Gemella	21,026	2.68	Prevotella_nanceiensis	11,751	2.54
Deinococcota	86	0.01	Micrococcaceae	23,233	2.51	Porphyromonas	19,657	2.5	Streptococcus_salivarius	11,652	2.52

## Data Availability

The raw data, the study protocol, the statistical analysis plan, the informed consent forms and other documents of this study are available from the corresponding author upon request due to ethical and privacy considerations involving sensitive human participant data.

## Supplementary Materials

The following supporting information can be downloaded at https://www.mdpi.com/article/10.3390/pathogens15030290/s1. Supplementary File S1 (Appendixes.pdf): Appendix S1: Participant Medical History Form (Greek and English versions); Appendix S2: Information Sheet for Participation in the Research Project (Greek and English versions); Appendix S3: Informed Consent Form (Greek and English versions). Supplementary File S2 (Abundances_All_Levels.xlsx): Read counts and relative abundance (%) across all taxonomic levels for the study cohort. Supplementary File S3 (Species_OTU_per_Sample.xlsx): Species-level read counts per participant. Supplementary File S4 (Indices_and_Demographics.xlsx): Shannon and Inverse Simpson indices per participant with basic demographic data.

## Abbreviations

The following abbreviations are used in this manuscript:

NGS	Next-Generation Sequencing
HMP	Human Microbiome Project
MetaHIT	Metagenomics of the Human Intestinal Tract
PPIs	Proton Pump Inhibitors
H2	Histamine-2 (receptor)
GIST	Gastrointestinal Stromal Tumor
IBD	Inflammatory Bowel Disease
OTUs	Operational Taxonomic Units
