# Supplementary material for: Characterization of the Gastric Antrum Microbiome in Helicobacter pylori-Negative Individuals: Insights from a Greek Population Using 16S rRNA Next-Generation Sequencing
Source: Pathogens. 2026 Mar 6;15(3):290. doi: 10.3390/pathogens15030290 (PMC13029027; doi:10.3390/pathogens15030290)
Supplement: Supplementary file 1 [file pathogens-15-00290-s001.zip › KAVVADA_APPENDIXES.pdf]

APPENDIXES

ΠΑΡΑΡΤΗΜΑ 1: ΙΣΤΟΡΙΚΟ ΣΥΜΜΕΤΕΧΟΝΤΑ

Το μικροβίωμα του άντρου του στομάχου και η συσχέτιση του με το H. pylori: ερευνητική μελέτη σε ασθενείς ελληνικού τριτοβάθμιου νοσοκομείου

Ημερομηνία:
Κωδικός ασθενούς:

ΙΣΤΟΡΙΚΟ ΣΥΜΜΕΤΕΧΟΝΤΑ

Φύλο:

Ηλικία:

Καταγωγή:

Κατανάλωση αλκοόλ:

Κάπνισμα:

Αιτία γαστροσκόπησης:

Κλινική εικόνα:

Λήψη φαρμάκων:

- ☐ Λήψη αναστολέων αντλίας πρωτονίων – PPIs ή άλλων αντιόξινων
- ☐ Λήψη αντιβιοτικών per os ή iv
- ☐ Λήψη προβιοτικών
- ☐ Λήψη βλεννογονοπροστατευτικών φαρμάκων
- ☐ Λήψη άλλων φαρμάκων:

Παρελθούσα λοίμωξη από H.pylori (ΝΑΙ / ΟΧΙ)

Λήψη θεραπείας από H.pylori (ΝΑΙ / ΟΧΙ)

Έναρξη θεραπείας:
Λήξη θεραπείας:

Ιστορικό προηγούμενων γαστροσκοπήσεων:

Ιστορικό πεπτικού έλκους:

Ιστορικό CA στομάχου-γαστρικού αδενωματος-MALT λεμφώματος:

Ιστορικό άλλης νόσου πεπτικού:

English Translation

APPENDIX 1: PARTICIPANT HISTORY

Gender:

Age:

Origin:

Alcohol consumption:

Smoking:

Reason for gastroscopy:

Clinical presentation:

Medication use:

- ☐ Use of proton pump inhibitors (PPIs) or other antacids:
- ☐ Use of antibiotics (per os or IV):
- ☐ Use of probiotics:
- ☐ Use of mucosal protective drugs:
- ☐ Use of other medications:

Previous H. pylori infection (YES / NO):

Received H. pylori eradication therapy (YES / NO):

Start of therapy:

End of therapy:

History of previous gastroscopies:

History of peptic ulcer disease:

History of gastric cancer / adenoma / MALT lymphoma:

History of other gastrointestinal diseases:

ΠΑΡΑΡΤΗΜΑ 2: ΈΝΤΥΠΟ ΕΝΗΜΕΡΩΣΗΣ ΓΙΑ ΤΗ ΣΥΜΜΕΤΟΧΗ ΣΕ ΕΡΕΥΝΗΤΙΚΟ ΕΡΓΟ

ΔΙΕΘΝΕΣ ΠΑΝΕΠΙΣΤΗΜΙΟ ΤΗΣ ΕΛΛΑΔΟΣ

Έντυπο ενημέρωσης για τη συμμετοχή σε ερευνητικό έργο

Ελληνικό Κείμενο

<u>Τίτλος έρευνας</u>: Το μικροβίωμα του άντρου του στομάχου και η συσχέτιση του με το H. pylori: πειραματική μελέτη σε ασθενείς ελληνικού τριτοβάθμιου νοσοκομείου

Το παρόν φυλλάδιο αποσκοπεί στην αναλυτική σας ενημέρωση επί της διδακτορικής έρευνας που θα πραγματοποιηθεί με τη συνεργασία των εξής φορέων:

1. Εργαστήριο Μικροβιολογίας, Τμήμα Βιοϊατρικών Επιστημών, ΔΙ.ΠΑ.Ε.
2. Εργαστήριο Μικροβιολογίας, Τμήμα Ιατρικής, Α.Π.Θ
3. Εργαστήριο ενδοσκοπήσεων του τμήματος Γαστρεντερολογίας, Α' Προπαιδευτικής Παθολογικής Κλινικής, Π.Γ.Ν.Θ. Α.Χ.Ε.Π.Α.

Σκοπός της παρούσας ερευνητικής μελέτης είναι να μελετηθεί το μικροβίωμα του άντρου του στομάχου σε εθελοντές (ασθενείς και μη) και να καταγραφούν πιθανές μεταβολές του μικροβιώματος σε ασθενείς με ενεργό λοίμωξη από ελικοβακτηρίδιο του πυλωρού (H. Pylori). Για να πραγματοποιηθεί ο στόχος, είναι αναγκαία η λήψη τεσσάρων (4) βιοψιών από το άντρο και το σώμα του στομάχου. Η λήψη των βιοψιών θα πραγματοποιηθεί στο ενδοσκοπικό εργαστήριο του τμήματος Γαστρεντερολογίας της Α΄ Προπαιδευτικής Παθολογικής Κλινικής του Πανεπιστημιακού Γενικού Νοσοσκομείου ΑΧΕΠΑ από ειδικό γαστρεντερολόγο, σε ελεγχόμενο και ασφαλή νοσοκομειακό περιβάλλον, στα πλαίσια του προγραμματισμένου ραντεβού σας για ενδοσκοπικό έλεγχο (γαστροσκόπηση). Συνεπώς, η διεξαγωγή επιπλέον γαστροσκοπήσεων για τις ανάγκες της έρευνας δεν κρίνεται αναγκαία.

Αυτή είναι μια εθελοντική συμμετοχή από την οποία μπορείτε να αποχωρήσετε ανά πάσα στιγμή. Οι συμμετέχοντες δεν αντιμετωπίζουν κανέναν κίνδυνο σχετικά με τη συλλογή δειγμάτων και κανένα κίνδυνο στη μετέπειτα εξέταση του βιολογικού απορριφθέντος υλικού τους. Δεν υπάρχουν αμοιβές ή έξοδα για τους συμμετέχοντες. Τα δείγματα πρόκειται να αναλυθούν ανώνυμα από τους ερευνητές και τα αποτελέσματα των μελετών θα αποθηκευτούν με ασφάλεια σε υπολογιστή και βιβλίο, με περιορισμένη πρόσβαση ερευνητών. Αυτά τα αποτελέσματα πρόκειται να χρησιμοποιηθούν για όσο διάστημα η έρευνα βρίσκεται σε εξέλιξη και στη συνέχεια θα αποθηκευτούν, με τα δεδομένα σας, με ασφάλεια και ανώνυμα για μελλοντικές μελέτες. Μπορείτε να αποκτήσετε πρόσβαση στα προσωπικά σας δεδομένα ή να αποσύρετε τα δείγματά σας από το έργο επικοινωνώντας με τον επόπτη. Σε περίπτωση νέων ευρημάτων, τα αποτελέσματα πρόκειται να δημοσιευθούν σε επιστημονικά περιοδικά ή να παρουσιαστούν σε συνέδρια.

<u>Επικοινωνία</u>

Ασημούλα Καββαδά, υποψήφια διδάκτωρ, τμήμα Βιοϊατρικών Επιστημών, Διεθνές Πανεπιστήμιο Ελλλάδος, τηλ. 6972109285, email: asimoula_444@yahoo.gr

Χατζηδημητρίου Μαρία, Καθηγήτρια Ιατρικής Βιοπαθολογίας - Ιατρικής Μικροβιολογίας - Ιατρικής Ανοσολογίας, Πρόεδρος του τμήματος Βιοϊατρικών Επιστημών, ΔΙ.ΠΑ.Ε,:

Υπεύθυνη Ερευνητικού Έργου, τηλ. 6944208095, email: chdimitr@ihu.gr

Ενημέρωση

Κατανοώ πλήρως τη φύση αυτής της έρευνας, έχω ενημερωθεί για τις λεπτομέρειες του συγκεκριμένου έργου και είχα την ευκαιρία να απαντήσω στις ερωτήσεις μου.

ΟΝΟΜΑΤΕΠΩΝΥΜΟ ΥΠΟΓΡΑΦΗ

English Translation

APPENDIX 2: INFORMATION SHEET FOR PARTICIPATION IN RESEARCH PROJECT

INTERNATIONAL HELLENIC UNIVERSITY

Information Sheet for Participation in Research Project

Title of the study: The antral gastric microbiome and its association with H. pylori: an experimental study in patients of a tertiary Greek hospital

This leaflet aims to provide you with detailed information about the doctoral research conducted in collaboration with the following institutions:

1. Microbiology Laboratory, Department of Biomedical Sciences, International Hellenic University (IHU)
2. Microbiology Laboratory, School of Medicine, Aristotle University of Thessaloniki (AUTH)
3. Endoscopy Unit, Department of Gastroenterology, 1st Propaedeutic Department of Internal Medicine, AHEPA University General Hospital

The purpose of this research study is to investigate the gastric antrum microbiome in volunteers (patients and non-patients) and to record potential changes in the microbiome of patients with active Helicobacter pylori infection. To achieve this objective, four (4) biopsies will be taken from the antrum and body of the stomach. Biopsies will be performed at the Endoscopy Unit of the Department of Gastroenterology, 1st Propaedeutic Department of Internal Medicine, AHEPA University General Hospital, by a specialized gastroenterologist in a controlled and safe hospital environment during your scheduled endoscopic appointment (gastroscopy). Therefore, no additional gastroscopies are required for the purposes of this study.

Participation in this study is entirely voluntary, and you may withdraw at any time. Participants are not exposed to any risk from sample collection or from subsequent examination of their discarded biological material. There are no fees or payments for participants. The samples will be analyzed anonymously by the researchers, and study results will be securely stored in

computer and in written form, with limited researcher access. These results will be used while the study is ongoing and will be securely and anonymously stored afterward for potential future research. You may access your personal data or withdraw your samples from the study by contacting the supervisor. If new findings arise, the results will be published in scientific journals or presented at conferences.

Contact

Asimoula Kavvada, PhD candidate, Department of Biomedical Sciences, International Hellenic University, tel. +30 6972109285, email: asimoula_444@yahoo.gr

Maria Chatzidimitriou, Professor of Medical Pathology – Medical Microbiology – Medical Immunology, Chair of the Department of Biomedical Sciences, IHU

Principal Investigator, tel. +30 6944208095, email: chdimitr@ihu.gr

Declaration

I fully understand the nature of this study, I have been informed about its details, and I have had the opportunity to ask any questions I may have.

FULL NAME SIGNATURE

ΠΑΡΑΡΤΗΜΑ 3: ΈΝΤΥΠΟ ΣΥΓΚΑΤΑΘΕΣΗΣ ΚΑΤΟΠΙΝ ΕΝΗΜΕΡΩΣΗΣ

ΔΙΕΘΝΕΣ ΠΑΝΕΠΙΣΤΗΜΙΟ ΤΗΣ ΕΛΛΑΔΟΣ
ΕΠΙΤΡΟΠΗ ΗΘΙΚΗΣ ΚΑΙ ΔΕΟΝΤΟΛΟΓΙΑΣ ΤΗΣ ΕΡΕΥΝΑΣ

Έντυπο Συγκατάθεσης Κατόπιν Ενημέρωσης

Καλείστε να συμμετέχετε σε μια έρευνα που διεξάγεται από την Ασημούλα Καββαδά, υποψήφια διδάκτωρ του τμήματος Βιοϊατρικών Επιστημών του ΔΙ.ΠΑ.Ε. Πρέπει να είστε τουλάχιστον 18 χρονών για να συμμετέχετε στην έρευνα. Η συμμετοχή σας είναι εθελοντική. Μπορείτε να αφιερώσετε όσο χρόνο χρειάζεστε για να διαβάσετε το «Έντυπο Συγκατάθεσης Κατόπιν Ενημέρωσης». Μπορείτε επίσης να αποφασίσετε να το συζητήσετε με την οικογένεια ή τους φίλους σας. Αντίγραφο αυτού του εντύπου θα σας δοθεί.

1. Σκοπός της έρευνας

Σας ζητάμε να συμμετέχετε στην παρούσα έρευνα γιατί ο σκοπός της είναι να καταγραφεί το μικροβίωμα του στομάχου και να αναδειχθούν οποιεσδήποτε μεταβολές του μικροβιώματος σε ασθενείς με μόλυνση ή και λοίμωξη από *H. pylori* σε σχέση με άτομα ελεύθερου ιστορικού.

Οι απαντήσεις στις ερωτήσεις της συνέντευξης και η συναίνεση λήψης βιοψιών κατά τη γαστροσκόπηση αποτελούν συγκατάθεση συμμετοχής στην παρούσα έρευνα.

2. Ενδεχόμενοι κίνδυνοι

Δεν υπάρχουν προβλέψιμοι κίνδυνοι που προκύπτουν από τη συμμετοχή σας στην παρούσα έρευνα. Εάν αισθανθείτε δυσφορία κατά την απάντησή σας σε συγκεκριμένες ερωτήσεις, παρακαλούμε μη διστάσετε να ζητήσετε να παραλειφθούν.

3. Ενδεχόμενα οφέλη για τα άτομα και την κοινωνία

Άμεσα οφέλη σε προσωπικό επίπεδο:
- Συμμετοχή στην έρευνα συνεπάγεται και τη διεξαγωγή καλλιεργειών του *H.pylori*.
- Η πλήρης καταγραφή του μικροβιώματος του στομάχου κάθε συμμετέχοντα.

Απώτερα οφέλη στο γενικό σύνολο: Η συσχέτιση της μεταβολής του μικροβιώματος με τις απώτερες επιπλοκές (καρκίνος στομάχου) που οφείλονται στη λοίμωξη του H. pylori, πιθανώς να οδηγήσουν σε ανεύρεση μέτρων πρόληψης.

4. Αποζημίωση για τη συμμετοχή

Δεν θα έχετε κάποιο άμεσο ή μελλοντικό σημαντικό οικονομικό όφελος από τη συμμετοχή σας στην παρούσα έρευνα.

5. Ενδεχόμενη σύγκρουση συμφερόντων

Κατά τη δήλωση των ερευνητών της έρευνας δεν υπάρχει σύγκρουση συμφερόντων από τη διεξαγωγή της παρούσας έρευνας.

6. Εμπιστευτικότητα

Οποιεσδήποτε πληροφορίες αποκτηθούν σχετικά με την παρούσα έρευνα, οι οποίες θα μπορούσαν να σας ταυτοποιήσουν προσωπικά, θα παραμείνουν απόρρητες και θα αποκαλυφθούν μόνο με την άδειά σας ή όπως προβλέπεται από τον νόμο. Οι πληροφορίες εκείνες που σας ταυτοποιούν προσωπικά, θα διατηρηθούν ξεχωριστά από τα υπόλοιπα δεδομένα που σας αφορούν. Τα δεδομένα θα φυλάσσονται με ευθύνη του ερευνητή. Σε περίπτωση ηχογράφησης, θα ζητηθεί η συγκατάθεσή σας. Μπορείτε να αρνηθείτε να ηχογραφηθείτε. Ο ερευνητής θα μεταγράψει τις ηχογραφήσεις και μπορεί να σας προμηθεύσει με ένα αντίγραφο του απομαγνητοφωνημένου κειμένου κατόπιν αιτήσεώς σας. Έχετε το δικαίωμα να ελέγξετε και να επεξεργαστείτε την απομαγνητοφώνηση. Προτάσεις οι οποίες έχετε ζητήσει από τον ερευνητή να παραληφθούν δεν θα χρησιμοποιηθούν και θα σβηστούν από όλα τα αντίστοιχα αρχεία. Σε περίπτωση που τα αποτελέσματα της έρευνας δημοσιευτούν ή παρουσιαστούν σε συνέδρια δεν θα συμπεριληφθούν πληροφορίες που θα αποκαλύπτουν την ταυτότητά σας. Σε περίπτωση που φωτογραφίες σας, βίντεο ή ακουστικές ηχογραφήσεις χρησιμοποιηθούν για εκπαιδευτικούς σκοπούς, η ταυτότητά σας θα προστατεύεται ή θα συγκαλύπτεται.

Σύμφωνα με το άρθρο 5 στοιχ.ε του Κανονισμού 2016/679 (GDPR), τα δεδομένα προσωπικού χαρακτήρα μπορούν να αποθηκεύονται για διάστημα μεγαλύτερο αυτού κατά το οποίο διεξάγεται η ερευνητική διαδικασία, εφόσον εφαρμόζονται τα κατάλληλα τεχνικά και οργανωτικά μέτρα που απαιτεί ο Κανονισμός 2016/679 (GDPR) για τη διασφάλιση των δικαιωμάτων και ελευθεριών του υποκειμένου (ή των υποκειμένων) των δεδομένων που έχουν συλλεχθεί στο πλαίσιο της έρευνας. Σε αυτήν τη βάση, τα δεδομένα της παρούσας έρευνας θα διατηρηθούν για τρία χρόνια μετά το πέρας αυτής.
Επιπρόσθετα, όσον αφορά στην ανάγκη περαιτέρω (δευτερογενούς) επεξεργασίας των προσωπικών δεδομένων, σύμφωνα με το άρθρο 5 στοιχ. β του Κανονισμού 2016/679 (GDPR), για λόγους ερευνητικούς, η δευτερογενής επεξεργασία των προσωπικών δεδομένων θεωρείται συμβατή με τους αρχικούς σκοπούς της επεξεργασίας χωρίς να γίνεται λόγος περί της ανάγκης λήψης τεχνικών και οργανωτικών μέτρων. Κατά συνέπεια, τα προσωπικά δεδομένα της παρούσας έρευνας δύνανται να χρησιμοποιηθούν και από άλλες έρευνες που θα εγκριθούν αρμοδίως χωρίς να χρειαστεί να δοθεί εκ νέου η συγκατάθεση των συμμετεχόντων.

7. Συμμετοχή και αποχώρηση

Μπορείτε να επιλέξετε να συμμετέχετε ή όχι στην παρούσα έρευνα. Αν συμμετέχετε εθελοντικά σε αυτή την έρευνα, μπορείτε να αποχωρήσετε οποιαδήποτε στιγμή χωρίς καμία συνέπεια. Μπορείτε επίσης να αρνηθείτε να απαντήσετε σε οποιεσδήποτε ερωτήσεις δεν επιθυμείτε να απαντήσετε και να παραμείνετε στην έρευνα. Ο ερευνητής μπορεί να σας ζητήσει να αποσυρθείτε από την έρευνα, αν ανακύψουν περιστάσεις που το απαιτούν.

8. Δικαιώματα των συμμετεχόντων στην έρευνα

Μπορείτε να αποσύρετε τη συγκατάθεσή σας οποιαδήποτε στιγμή και να διακόψετε τη συμμετοχή σας χωρίς να υποστείτε καμία κύρωση.

9. Ταυτότητα των ερευνητών

Αν έχετε οποιεσδήποτε ερωτήσεις ή ανησυχίες σε σχέση με την έρευνα, μη διστάσετε να επικοινωνήσετε με τον ερευνητή:

Ασημούλα Καββαδά
email επικοινωνίας: asimoula_444@yahoo.gr
τηλέφωνο επικοινωνίας: 6972109285

Διάβασα τα παραπάνω και αποδέχομαι τη συμμετοχή μου στην έρευνα.

Ονοματεπώνυμο____________________ Υπογραφή___________________________________

Ημερομηνία____

APPENDIX 3: INFORMED CONSENT FORM

INTERNATIONAL HELLENIC UNIVERSITY
RESEARCH ETHICS AND DEONTOLOGY COMMITTEE

Informed Consent Form

You are invited to participate in a research study conducted by Asimoula Kavvada, a PhD candidate at the Department of Biomedical Sciences of the International Hellenic University. You must be at least 18 years old to participate. Your participation is voluntary. Please take as much time as you need to read this 'Informed Consent Form'. You may also discuss it with your family or friends. A copy of this form will be given to you.

1. Purpose of the study
The purpose of this study is to record the gastric microbiome and to highlight any changes in patients with H. pylori infection compared to those without. By agreeing to answer the interview questions and to the collection of biopsy samples during gastroscopy, you consent to participate in this research.

2. Potential Risks
No foreseeable risks are expected from your participation in this research. If you feel discomfort when answering any questions, please request to skip them.

3. Potential Benefits
Personal benefits:

- Participation includes laboratory culture for H. pylori.
- Comprehensive recording of each participant's gastric microbiome.

General benefits:
The correlation between microbiome alterations and long-term complications such as gastric cancer may help identify preventive measures.

4. Compensation
You will not receive any direct or future financial benefit from participating in this study.

5. Conflict of Interest
The researchers declare no conflict of interest arising from the conduct of this study.

6. Confidentiality
All data collected that could personally identify you will remain confidential and will only be disclosed with your permission or as required by law. Personally identifiable information will be stored separately from other research data. Data will be securely stored by the researcher. If audio recordings are used, your consent will be requested. You may refuse to be recorded. Transcriptions can be provided upon request. Any parts you request to be omitted will be deleted from all files. If results are published or presented, your identity will not be revealed.

According to Article 5(e) of Regulation (EU) 2016/679 (GDPR), personal data may be retained for up to three years after the completion of the research under appropriate safeguards. Secondary use of anonymized data for future studies may occur without renewed consent.

7. Participation and Withdrawal

You may choose to participate or not. Participation is voluntary, and you may withdraw at any time without any consequence. You may also refuse to answer specific questions and remain in the study. The researcher may ask you to withdraw if necessary.

8. Rights of Participants

You may withdraw your consent at any time without penalty.

9. Researcher Contact Information

Asimoula Kavvada
Email: asimoula_444@yahoo.gr
Phone: 6972109285

I have read and understood the above information and consent to participate in this research.

Name: ____________________ Signature: ____________________
Date: ____________________
